# Strain relaxation in InAs heteroepitaxy on lattice-mismatched substrates

**DOI:** 10.1038/s41598-020-61527-9

**Published:** 2020-03-12

**Authors:** Akihiro Ohtake, Takaaki Mano, Yoshiki Sakuma

**Affiliations:** National Institute for Materials Science (NIMS), Tsukuba, 305-0044 Japan

**Keywords:** Structural properties, Structural properties

## Abstract

Strain relaxation processes in InAs heteroepitaxy have been studied. While InAs grows in a layer-by-layer mode on lattice-mismatched substrates of GaAs(111)A, Si(111), and GaSb(111)A, the strain relaxation process strongly depends on the lattice mismatch. The density of threading defects in the InAs film increases with lattice mismatch. We found that the peak width in x-ray diffraction is insensitive to the defect density, but critically depends on the residual lattice strain in InAs films.

## Introduction

Heteroepitaxy of semiconductors has opened up new possibilities for band-structure engineering and novel devices, including strained-layer structures. The flexibility in the choice of materials for the formation of heterostructures is often limited by lattice mismatch. Heteroepitaxy in lattice-mismatched systems usually follows a Stranski-Krastanov (SK) growth mode: a pseudomorphic two-dimensional layer is formed below a certain critical thickness, and is followed by the formation of three-dimensional islands. A prototypical example of such a system is Ge on Si (lattice mismatch ≈4.2%), in which layer-by-layer growth is limited to 3–4 monolayer (ML). The island formation is highly undesirable, because it prevents the growth of smooth films and introduces nucleation centers for defects. Thus, significant efforts have been devoted to suppress the strain-induced islanding and strain-relieving defects. It has been reported that the islanding in the Ge/Si system is effectively suppressed by introducing As and Sb as surfactant species^1–3^.

The SK growth occurs also in the InAs/GaAs(001) system having lattice mismatch of 7.2%: InAs islands are formed at the film thickness of 1.6 ML^4^. On the other hand, the use of the (111)A-oriented GaAs substrates forces the InAs film to grow in a layer-by-layer mode^5–7^. The layer-by-layer growth of the (111)A-oriented InAs film is accompanied by the formation of a misfit dislocation network at the InAs/GaAs interface^6^, so that the generation of defects in the film is strongly suppressed. Similar strain relaxation has been reported for GaSb/GaAs(001) heteroepitaxy, which is known for so-called interfacial misfit array growth^8^.

The novel growth technique has been successfully applied to the layer-by-layer growth of InAs on Si(111)^9^, GaSb growth on InAs/Si(111)^10^, and to the improvement of the crystalline quality of InGaAs^11^ and GaSb^12^ on InAs/GaAs(111)A. This technique has a great advantage, especially for the growth on Si(111), because the formation of antiphase domain boundaries in InAs films is suppressed, in contrast with the growth on the (001)-oriented substrate. However, strain relaxation processes of InAs are far from being completely understood, and the crystalline quality of InAs has not been studied in detail.

This paper reports the strain relaxation processes and structural properties of InAs films heteroepitaxially grown on the lattice-mismatched substrates of GaAs(111)A, Si(111), and GaSb(111)A. While the InAs(111)A film grows in a layer-by-layer mode on all substrates irrespective of the lattice mismatch, the strain relaxation process behaves differently depending on the lattice mismatch. Fully-strained pseudomorphic InAs layers continue to grow above ~50 ML on the nearly lattice matched GaSb substrates (lattice mismatch of −0.61%), while in the InAs/Si system with the largest lattice mismatch of 11.4%, mostly-relaxed InAs films are formed even at the very initial stage of the growth. The in-plane compressive strain in InAs on GaAs (lattice mismatch of 7.2%) gradually relaxed as the growth proceeds, but the strain is not fully relaxed even in the 100nm-thick InAs film. Our x-ray diffraction (XRD) measurements revealed that the residual strain in InAs film is responsible for the peak broadening in XRD profiles. On the other hand, the peak width of x-ray rocking curve is insensitive to the density of threading defects.

## Results and discussion

**Figure 1:**
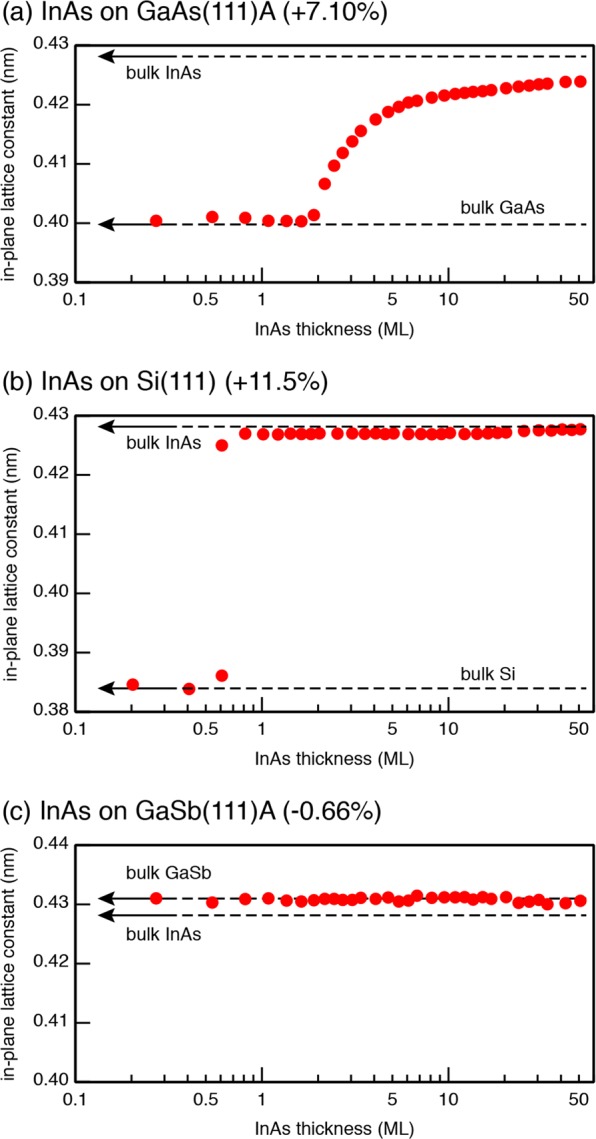
The variation of the in-plane lattice constant (*d*_110_) of InAs films grown on the GaAs(111)A (**a**), Si(111) (**b**), and GaSb(111)A (**c**) substrates. The values were measured from the distance between the 11 and $$\bar{1}$$$$\bar{1}$$ reflections in the RHEED patterns.

  Figure 1(a) shows the variation in the in-plane lattice constant (*d*_110_) of the InAs film growing on the GaAs(111)A substrate. The *d*_110_ values were measured from the distance between the 11 and $$\bar{1}$$$$\bar{1}$$ reflections in reflection high-energy electron diffraction (RHEED) patterns along the [$$\bar{1}$$$$\bar{1}$$2] direction. The data clearly shows that InAs pseudomorphically grows below ~2 ML, and that the in-plane lattice constant gradually increases with film thickness above ~2 ML, in good agreement with earlier results^6,7^. The strain in the InAs film has relaxed by only ~80%, even after the 50 ML-growth.

Similarly to the case for InAs/GaAs(111)A, the InAs film is two-dimensionally grown on the In-terminated Si(111) substrate^9^. However, the strain relaxation processes between the two systems are quite different. Shown in Fig. 1(b) is the variation in the *d*_110_ value for the InAs growth on the Si(111) substrate. A new set of streaks from the InAs film appeared in the RHEED patterns at the very early stage of the growth (~0.6 ML), in addition to those from the Si substrate. The spacing of streaks is quite close to the value of bulk InAs, and remains almost unchanged throughout the growth (<50 ML). This indicates that, on the Si(111) substrate, the InAs film was nucleated with its inherent lattice constant, and that pseudomorhic InAs layers are not formed. Thus, it is likely that the lattice mismatch of InAs/Si (11.5%) is too large to be accommodated by elastic deformation of thin InAs films.

In the nearly lattice-matched system of InAs on GaSb(111)A (lattice mismatch ≈0.61%), as shown in Fig. 1(c), no significant change in the *d*_110_ value is observed below 50 ML (17.5 nm). Thus, it is plausible that 50 ML-InAs films are coherently strained to the GaSb substrate. This is consistent with the critical thickness (~20 nm) for the onset of misfit dislocations in InAs on GaSb estimated on the basis of the model proposed by Matthews and Blakeslee^13^.

**Figure 2:**
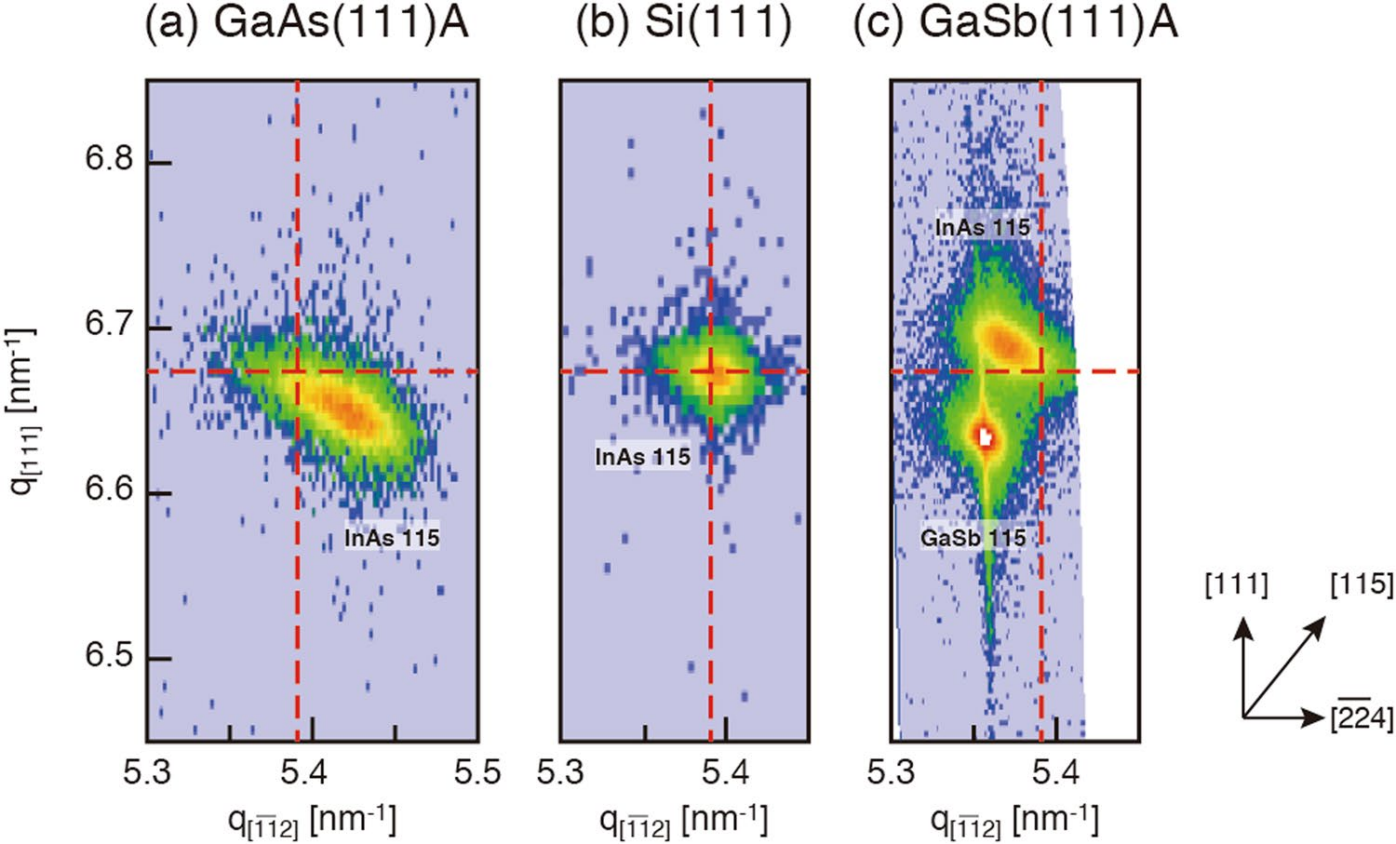
Reciprocal-space maps (RSMs) of the asymmetric 115 reflection measured from 100 nm-InAs films on GaAs (**a**), Si (**b**), and GaSb (**c**) substrates. The dashed lines show the position of bulk InAs. The RSMs were generated using the Bruker DIFFRAC.LEPTOS software package ver. 7.7, and were assembled using the Adobe Illustrator CS6.

Figure 2(a,b) show the two-dimensional reciprocal-space maps (RSMs) for the asymmetric 115 reflection of 100 nm-thick InAs films grown on the GaAs(111)A and In-terminated Si(111) substrates, respectively. The vertical and horizontal axes correspond to the indices along the [111] and [$$\bar{1}$$$$\bar{1}$$2] directions, respectively. The in-plane lattice constant of InAs grown on GaAs is 0.4236 nm, and is quite smaller than that of bulk InAs (0.4284 nm). The residual strain in the InAs film (*d*_110_ (film) − *d*_110_(bulk))/*d*_110_(bulk)) is −1.124%. According to classical elastic theory, the in-plane compressive strain causes the expansion of the lattice constant, *d*_111_, in the direction normal to the surface. Poisson’s ratio *σ* of the strained InAs(111)A layer is *σ* = (*c*_11_ + 2*c*_12_ + 4*c*_44_)/(2*c*_11_ + 4*c*_12_ − 4*c*_44_) = 1.75, where *c*_11_ = 8.33, *c*_12_ = 4.53, and *c*_44_ = 3.96 (× 10^6^ Pa) are the elastic stiffness coefficients of InAs^14,15^. Thus, using the measured *d*_110_ value, the *d*_111_ value is estimated to be 0.3520 nm, in good agreement with the measured value of 0.3511 nm.

The *d*_110_ and *d*_111_ values of InAs grown on Si(111) are 0.4281 and 0.3499 nm, which are quite close to those of bulk InAs (0.4284 nm and 0.3498 nm), as seen in Fig. 2(b). The residual in-plane strain is estimated to be −0.089%, indicating that the InAs film is mostly relaxed.

In contrast to the case for the compressively strained systems of InAs/GaAs and InAs/Si, InAs films are tensile strained on GaSb. The measured *d*_110_ (0.4303 nm) and *d*_111_ (0.3489 nm) values of the InAs film are slightly larger and smaller, respectively, than those of bulk values. The tensile in-plane strain in InAs is relaxed by ~30%, indicating that the strain begins to relax in the InAs film below 100 nm. This is broadly consistent with the mechanical equilibrium model^13^, from which the critical thickness is estimated to be 20 nm, as mentioned earlier. On the other hand, the growth experiments on the (001)-oriented GaSb substrate showed that InAs films as thick as 200 nm are fully strained^16^. It is suggested that the strain relaxation mechanism is a strong function of substrate orientation.

**Figure 3:**
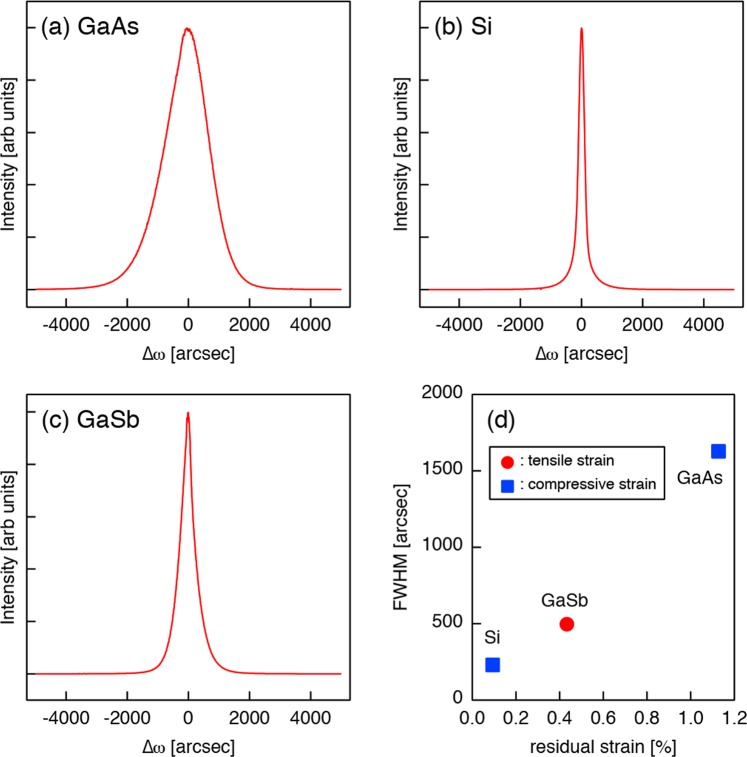
111 XRCs of 100 nm-InAs films grown on GaAs(111)A (**a**), Si(111) (**b**), and GaSb(111)A (**c**). (**d**) FWHM values of the 111 XRCs plotted as a function of residual strain.

Figure 3(a–c) compare x-ray rocking curves (XRCs) measured from 100nm-InAs on GaAs(111)A, Si(111), and GaSb(111)A. The FWHM values are 1627.7 arcsec (a), 230.05 arcsec (b), and 496.83 arcsec (c). It is well known that crystal imperfections, such as mosaic domains, threading defects, structural inhomogeneities, and lattice distortions, cause a broadening of XRD profiles. However, it is difficult to specify the origin of the peak broadening from the rocking curves of the symmetric 111 reflection alone. On the other hand, RSMs of asymmetric reflections provides important information of the structural quality of the InAs film; as can be seen in Fig. 3(a), the 115 reflection has an elliptical shape, which is elongated along the direction perpendicular to the [115] azimuth. This means that the broadening of XRD peaks arises from the misorientation of InAs lattice planes. While a slight broadening is observed in Fig. 2(c), such a broadening is not observed in Fig. 2(b). In Fig. 3(d), the FWHM values are plotted as a function of the absolute value of residual strain: the FWHM value increases with the absolute value of residual strain. In general, uniformly-strained flat layers are unstable against the modulation of the surface profile, which allows a partial relaxation of the strain by elastic deformation^17–19^. Thus, it is likely that the strained InAs film on the GaAs(111)A substrate is elastically deformed to accommodate the residual strain. Such elastic deformation is accompanied by the misorientation of the InAs lattice planes, resulting in the broadening perpendicular to the reciprocal lattice vector of the corresponding diffraction spot.

**Figure 4:**
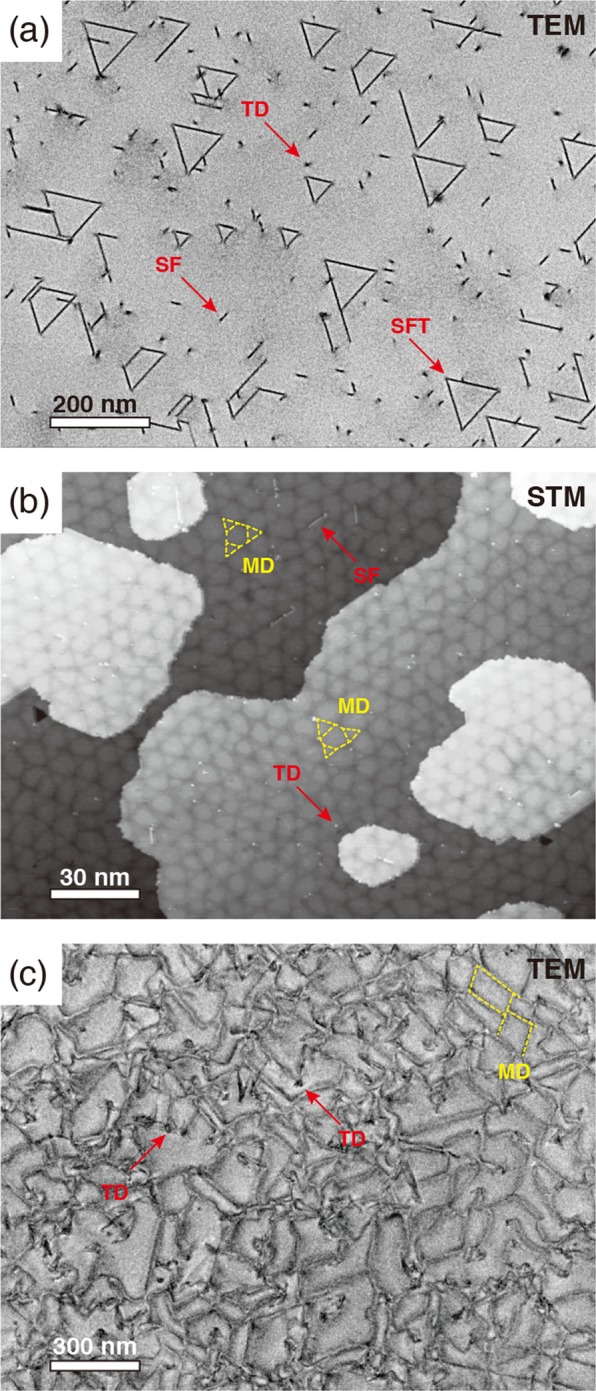
Plan-view TEM images of InAs films grown on Si(111) (**a**) and GaSb(111)A (**c**) substrates. (**b**) shows the scanning tunneling microscopy (STM) image of 4 ML-InAs on GaAs(111)A. Image dimensions of (**a–c**) are 900 nm × 1200 nm, 200 nm × 150 nm, and 1500 nm × 2000 nm, respectively. The STM image was acquired in the constant current mode with a tunneling current of 0.1 nA and a sample voltage of − 3 V. The arrows marked TD, SF, and SFT, indicate the threading dislocation, stacking fault, and stacking-fault tetrahedron, respectively, while the position of the misfit dislocation (MD) is indicated by the yellow dashed lines. We note that the Si substrate was completely removed from the sample (**a**), while the sample (**c**) consists of the InAs film and GaSb substrate.

Figure 4(a) shows a representative plan-view transmission electron microscopy (TEM) image of InAs on Si(111). A high density of threading defects, such as threading dislocations, stacking faults, and stacking-fault tetrahedra, is clearly observed. The densities of these defects are listed in Table 1. Similar TEM images were obtained for InAs on GaAs(111)A and GaSb(111)A with lower densities of threading defects. On the other hand, as mentioned earlier, the FWHM value of XRC for InAs/Si is much smaller than that for InAs/GaAs. The FWHM value of XRC is often cited as a measure of structural quality: it has been generally believed that the existence of high densities of threading defects causes the broadening of XRC width. However, the present results show that narrower (broader) peaks in XRCs are not necessarily an indication of lower (higher) density of threading defects in heteroepitaxial layers.

**Table 1:** Density of threading defects in InAs on GaAs(111)A, Si(111), and GaSb(111)A [cm^−2^].

	InAs/GaAs(111)A	InAs/Si(111)	InAs/GaSb(111)A
threading dislocation	2.7 × 10^9^	7.5 × 10^9^	1.7 × 10^9^
stacking fault	3.0 × 10^9^	5.1 × 10^9^	1.9 × 10^9^
stacking-fault tetrahedron	8.2 × 10^8^	3.0 × 10^9^	5.4 × 10^8^

Previous studies have shown that the two-dimensional growth of InAs on GaAs(111)A and Si(111) substrates is accompanied by the formation of a misfit dislocation network at the interfaces to accommodate the strain^6,7^. In the ideal case, perfect dislocation arrays are formed covering all the interface area, leaving the growing films free of threading dislocations. However, as shown in Fig. 4(b), the actual misfit dislocation arrays are not perfect; it is likely that the disorder in the misfit dislocation network acts as the source of the threading defects (arrows). The averaged period of misfit-dislocation network *p*_md_ is given by *p*_md_ = ***b***/*f*, where ***b*** is the Burgers vector and *f* is the lattice misfit. For partially-relaxed 100 nm-InAs on GaAs(111)A, the *p*_md_ value is estimated to be 6.7 nm, which is twice as large as that for the fully-relaxed interface of InAs/Si(111) (3.3 nm). If we assume that the nucleation probability of threading defects increases with the density of misfit dislocations, we can explain why the defect density is higher in the InAs film on Si(111).

In the nearly lattice-matched InAs/GaSb system, the densities of threading defects are slightly lower than those for InAs/GaAs, as shown in Table 1. On the other hand, since the lattice mismatch of InAs/GaSb is more than an order of magnitude smaller than that for InAs/GaAs, one may expect a much lower density of misfit dislocations. Figure 4(c) shows the plan-view TEM image of 100nm-InAs/GaSb(111)A; since the sample contains both the InAs film and the GaSb substrate, misfit dislocations at the interface are imaged (yellow dashed lines), as well as threading defects (red arrows). We note that only threading defects in the InAs films are imaged at thinner (<100 nm) regions (See Supplementary Fig. S1). As compared with InAs on GaAs (Fig. 4(b)), the distribution of the misfit dislocations is highly irregular; such an irregular configuration is likely to be responsible for the increase of threading defects: as indicated by arrows in Fig. 4(c), threading dislocations are often observed at the end of misfit dislocations. Thus, it is reasonable to consider that the extremely large critical thickness for the generation of misfit dislocation makes it difficult to rearrage highly separated misfit dislocations to an ordered periodic network at the buried interface beneath thick InAs layers.

## Conclusions

The effects of lattice mismatch on the growth mode and strain relaxation process in InAs heteroepitaxy on GaSb(111)A, GaAs(111)A, and Si(111) substrates have been studied. The highest and lowest defect densities are identified in the highly lattice-mismatched InAs/Si and the nearly lattice-matched InAs/GaSb systems, respectively. On the other hand, the peak width in XRD is insensitive to the lattice mismatch and is roughly proportional to the residual strain in InAs films.

## Methods

The growth experiments were carried out in a multi-chamber MBE system. The As-doped Si(111) wafer was cleaned by radiatively heating at 950 °C in the MBE chamber^20^. Clean and well-ordered (7 × 7) reconstructions were confirmed by STM, x-ray photoelectron spectroscopy, and RHEED. In-terminated (4 × 1) reconstruction was prepared by depositing 1 ML of In on the Si(111)-(7 × 7) surface at 450 °C. The clean surfaces of GaAs(111)A and GaSb(111)A were prepared by growing undoped homoepitaxial layers at 450 °C on the thermally cleaned substrates^21,22^. The GaAs (GaSb) layers were grown with an As_4_/Ga (Sb_4_/Ga) flux ratio of ~50 (~8). While only a (2 × 2) reconstruction is observed on the GaAs(111)A surface under the conventional MBE condition, (2 × 2) (2$$\sqrt{3}$$$$\times 2\sqrt{3}$$) and (1 × 5) reconstructions were observed on GaSb(111)A as the surface Sb coverage is increased. In the present study, GaAs(111)A-(2 × 2) and GaSb(111)A-(2$$\sqrt{3}$$$$\times 2\sqrt{3}$$) surfaces were used as substrates for the InAs growth.

InAs films were grown on the GaAs(111)A substrate at 450 °C with an As_4_/In flux ratio of ~50. On the other hand, the growth on Si(111) and GaSb(111)A results in the formation of twins and islands under otherwise identical condition. Thus, to suppress the growth front roughening, the growth on these substrates were carried out using As_2_ molecules having higher reactivity to In adatoms with a higher As_2_/In ratio of ~150. The growth rate of InAs was approximately 0.034 ML/s, which was calibrated by RHEED intensity oscillation measurements on the (001)-oriented InAs substrate. Here, 1 ML of InAs is defined as 6.3 × 10^14^ atoms/cm^2^, which is the site-number density of unreconstructed InAs(111)A surface. Great care was taken to avoid the possible adsorption of As molecules on Si(111) substrate for the growth on the In-terminated Si(111) substrate, because the In-terminated Si surface easily reacts with As molecules to transform itself to the As-terminated one, on which InAs grows in an island mode^9^. Thus, the InAs growth on Si is initiated under the lower As_2_/In ratio of 30, and the flux ratio is increased to ~150 after the 10 ML-growth. The growth process of InAs was monitored by RHEED in real time, and the structural properties have been characterized using XRD and TEM. High resolution XRD measurements were carried out using a monochromatic Cu K*α*1 radiation. A channel-cut analyzer crystal was used for XRC measurements. RSM data were obtained using an one-dimensional array detector. The density and type of threading defects in InAs films were assessed by TEM operated at 200 keV.

## Supplementary information

Supplementary Information.

## Data Availability

The data that support the findings of this study are available from the corresponding author upon reasonable request.
