# Supplementary material for: Strain relaxation in InAs heteroepitaxy on lattice-mismatched substrates
Source: Sci Rep. 2020 Mar 12;10:4606. doi: 10.1038/s41598-020-61527-9 (PMC7067763; doi:10.1038/s41598-020-61527-9)
Supplement: Supplementary file 1 — Supplementary Information. [file 41598_2020_61527_MOESM1_ESM.pdf]

Supplementary Information

Strain relaxation in InAs heteroepitaxy on lattice-mismatched substrates

Akihiro Ohtake,[*,†] *Takaaki Mano,*[†] *and Yoshiki Sakuma*[†]

[†]*National Institute for Materials Science (NIMS), Tsukuba 305-0044, Japan*

Corresponding Author: OHTAKE.Akihiro@nims.go.jp

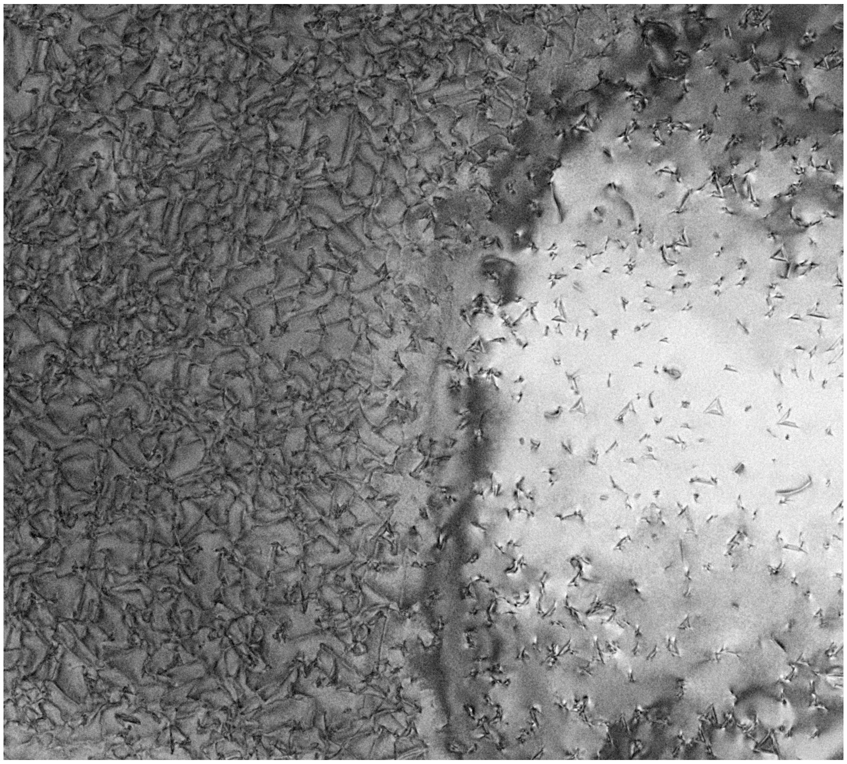

Figure S1. Plan-view TEM image of the 100 nm-InAs film grown on the GaSb(111)A substrate. The image dimension is 3600 x 4000 nm. In the thin (bright) region, threading dislocations, stacking faults, and stacking-fault tetrahedra are clearly observed, similar to the image for InAs/Si(111) (Fig. 4(a)), while misfit dislocations at the interface as well as threading defects are imaged in the thick (dark) region.
